# Seasonal Changes in the Prevalence of Hyperkalemia in the Emergency Department: A Single Center Study

**DOI:** 10.3390/medicina58020282

**Published:** 2022-02-14

**Authors:** Teppei Koyama, Ryuichiro Makinouchi, Shinji Machida, Katsuomi Matsui, Yugo Shibagaki, Naohiko Imai

**Affiliations:** St. Marianna University School of Medicine, Yokohama City Seibu Hospital, Yokohama 241-0811, Japan; teppei.koyama@marianna-u.ac.jp (T.K.); ryuichiro.makinouchi@marianna-u.ac.jp (R.M.); jnss0322@gmail.com (S.M.); m.katsuomi@marianna-u.ac.jp (K.M.); eugo@wc4.so-net.ne.jp (Y.S.)

**Keywords:** hyperkalemia, prevalence, seasonal, emergency department

## Abstract

*Background:* Hyperkalemia is an electrolyte disorder frequently encountered in the emergency department. There are few studies on seasonal variation in the prevalence of hyperkalemia. The aim of this study was to investigate the seasonal changes in the prevalence of hyperkalemia in the emergency department. *Materials and Methods:* We retrospectively reviewed a total of 24,085 patients presented to the emergency department between January 2012 and December 2020. Age, gender, serum potassium level, and serum creatinine level were recorded. The definition used for hyperkalemia was a serum potassium level of ≥ 5.5 mEq/L. Renal function was divided into two categories: preserved (eGFR ≥ 60 mL/min/1.73 m^2^) or reduced (eGFR < 60 mL/min/1.73 m^2^). *Results:* The prevalence of hyperkalemia was 2.1% in patients with preserved renal function and was 11.9% in patients with reduced renal function (*p* < 0.001). The prevalence of hyperkalemia was highest in winter, followed by spring, autumn, and summer in patients with preserved renal function (*p* < 0.001) and those with reduced renal function (*p* < 0.001). There was a linear correlation between monthly weather temperature and the prevalence of hyperkalemia in patients with preserved renal function (r = −0.392; *p* < 0.001) and those with reduced renal function (r = −0.487; *p* < 0.001). *Conclusions*: we found that the prevalence of hyperkalemia was significantly higher in winter for both patients with preserved renal function and those with reduced renal function.

## 1. Introduction

Hyperkalemia is an electrolyte disorder frequently encountered in the emergency department (ED). The prevalence of hyperkalemia in the ED has been well documented and depending on the definition of hyperkalemia, the prevalence of hyperkalemia in the ED has been reported to range from 0.3% to 13.3% [1,2,3,4,5]. The prevalence of various diseases, including infectious diseases, cardiovascular diseases, and kidney diseases are known to have seasonal variations [6,7,8,9].

Seasonal changes in the prevalence of fluid and electrolyte disorders, such as hypernatremia and hyponatremia are also known [10,11,12,13]. Regarding hyperkalemia, only a few studies reported seasonal variation in the prevalence of hyperkalemia in hemodialysis patients [14,15,16].

To the best of our knowledge, no study has reported the seasonal variation in the prevalence of hyperkalemia in patients with preserved renal function. The aim of this study was to evaluate the seasonal variation in the prevalence of hyperkalemia in patients with preserved renal function who visited the ED.

## 2. Materials and Methods

We conducted a cross-sectional study of all adult patients (18 years and older) whose serum potassium levels were measured in our ED between January 2012 and December 2020. The definition used for hyperkalemia was a serum potassium level of ≥5.5 mEq/L, as used in a previous study [17]. Age, sex, serum potassium level, and serum creatinine level were recorded. The first serum potassium level in the ED was used in this study. The estimated glomerular filtration rate (eGFR) of each patient was calculated using the following formula: eGFR (mL/min/1.73 m^2^) = 194 × serum creatinine^−1.094^ × age^−0.287^ × 0.739 (if female) [18]. Renal function was divided into two groups: preserved (eGFR ≥ 60 mL/min/1.73 m^2^) or reduced (eGFR < 60 mL/min/1.73 m^2^), as used in a previous study [19]. The temperature of the region was based on data provided by the Japan Meteorological Agency. The definition of the four seasons provided was as follows: spring: March–May; summer: June–August; autumn: September–November; winter: December–February. After obtaining ethical approval from the ethics committee of St. Marianna University (approval code: 5390, approval date: 3 September 2021), we conducted this clinical research in accordance with the principles of the Declaration of Helsinki.

### Statistics

Continuous variables were shown as medians and interquartile ranges, and categorical variables as percentages. The Mann–Whitney U test was used for the comparison of continuous variables and the χ^2^ test was used for comparison of the proportions between groups. A *p*-value of <0.05 was considered statistically significant. All data analyses in this study were performed using SPSS, Version 21.0 (IBM Corp, Armonk, NY, USA).

## 3. Results

### 3.1. Patients

This retrospective study included a total of 24,085 adult patients; 12,979 patients had preserved renal function and 11,106 patients had reduced renal function. Patients with preserved renal function had a median eGFR of 81 (70–98) mL/min/1.73 m^2^, and patients with reduced renal function had a median eGFR of 40 (22–51). The prevalence of hyperkalemia was 2.1% in patients with preserved renal function and 11.9% in patients with impaired renal function. The characteristics of the patients are summarized (Table 1).

### 3.2. Prevalence of Hyperkalemia According to Age and Renal Function

**Figure 1:**
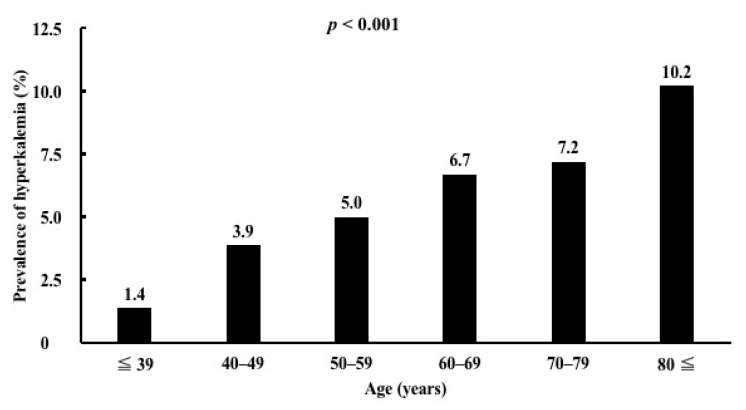
Prevalence of hyperkalemia according to age.

**Figure 2:**
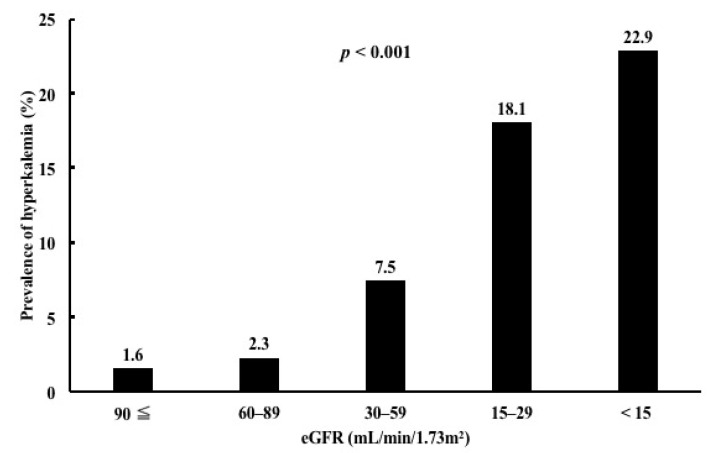
Prevalence of hyperkalemia according to renal function.

The prevalence of hyperkalemia according to age and renal function is shown. The prevalence of hyperkalemia was highest in patients older than 80 years and lowest in patients younger than 40 years (*p* < 0.001) (Figure 1). The prevalence of hyperkalemia was highest in patients with eGFR < 15 mL/min/1.73 m^2^ and lowest in patients with eGFR > 90 mL/min/1.73 m^2^ (*p* < 0.001) (Figure 2).

### 3.3. Seasonal Variation in the Prevalence of Hyperkalemia

The prevalence of hyperkalemia in each season is shown (Table 2). In patients with preserved renal function, the prevalence of hyperkalemia was highest in winter followed by spring, autumn, and summer (*p* < 0.001 vs. summer and autumn). In patients with reduced renal function, the prevalence of hyperkalemia was highest in winter followed by spring, autumn, and summer (*p* < 0.001 vs. summer and autumn).

### 3.4. Prevalence of Hyperkalemia and Monthly Weather Temperature

**Figure 3:**
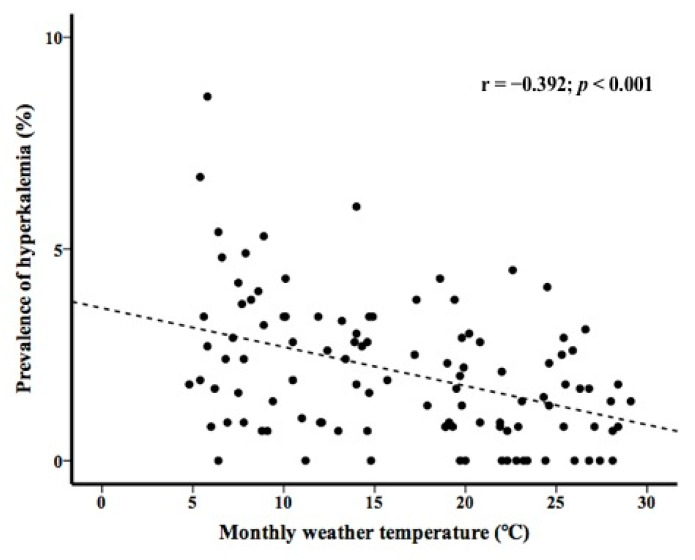
Prevalence of hyperkalemia and monthly weather temperature in patients with preserved renal function.

**Figure 4:**
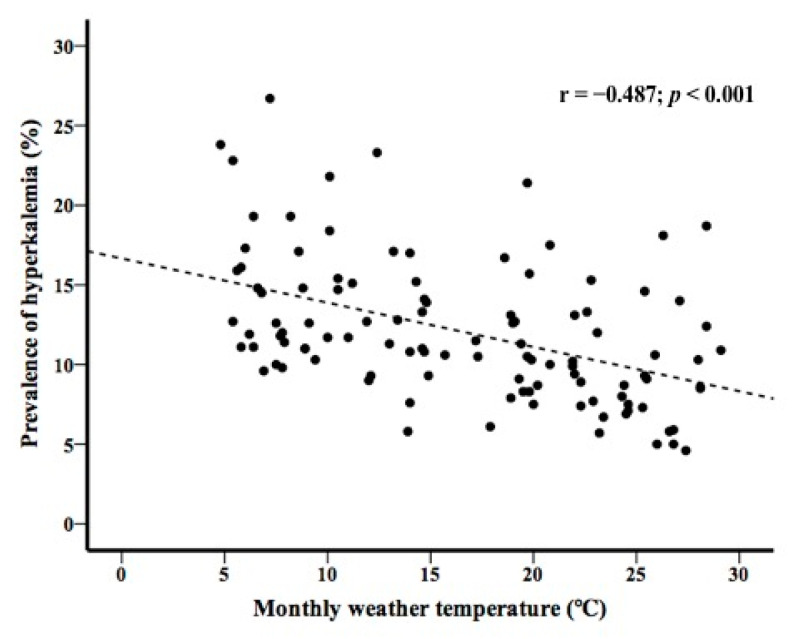
Prevalence of hyperkalemia and monthly weather temperature in patients with reduced renal function.

In patients with preserved renal function, a linear relationship between the prevalence of hyperkalemia and monthly weather temperature was observed (r = −0.392; *p* < 0.001) (Figure 3). Similarly, in patients with reduced renal function, a linear relationship between the prevalence of hyperkalemia and monthly weather temperature was observed (r = −0.487; *p* < 0.001) (Figure 4).

## 4. Discussion

In this retrospective study, we showed that the prevalence of hyperkalemia in the ED was highest in winter, both in patients with preserved renal function and in those with reduced renal function. As for hyperkalemia, as far as we know, only a few studies have reported the seasonal prevalence of hyperkalemia in hemodialysis patients [14,15,16]. Our results were comparable to previous reports of a winter peak in serum potassium levels in hemodialysis patients [14,15,16].

Vegetables and fruits are the main sources of potassium, and its intake might be related to seasonal trends. Various results on the seasonality of fruit and vegetable intake have been reported from around the world, but since dietary habits vary widely from country to country, reports from other countries are likely not applicable [20,21,22]. Reports from Japan have shown seasonal variations in the intake of foods such as vegetables and fruits [23,24]. This reflects the Japanese lifestyle of eating watermelon in summer and mandarin oranges and apples in winter, while bananas and kiwis are eaten all year around. Also, in Japan, elderly people are reported to consume more fruit and vegetable than younger people [25]. Although it has been reported that the intake of fruits and green and yellow vegetable are higher in summer than in winter, the potassium content of fruits and vegetables varies widely from food to food and these factors need to be taken into account when considering the effect on serum potassium levels [26]. It has also been reported that in Japan, potatoes are consumed more in winter than in summer [23]. It is well known that fruits and vegetables are rich in potassium, but it is often forgotten that potatoes, a white vegetable, contain the highest amount of potassium [27]. Therefore, the effect of eating more potatoes in winter on serum potassium levels can-not be ignored. Finally, in hot environments, potassium is lost through sweat and urine, and the potassium balance may become negative [28]. These factors may also be responsible for the seasonal variation in the prevalence of hyperkalemia.

Serum aldosterone levels have been reported to fluctuate seasonally [29,30]. On the other hand, it is well known that aldosterone is involved in the homeostasis of serum potassium concentration [31]. Although the results of studies on seasonal variations in serum aldosterone levels are inconsistent, it is possible that seasonal variations in serum aldosterone levels might have influenced seasonal variations in the prevalence of hyperkalemia.

A strong association between monthly weather temperature and the prevalence of hyponatremia has been reported [10,12,17]. The present study shows a strong association between monthly weather temperature and the prevalence of hyperkalemia, similar to that reported for hyponatremia. Since the increase in mortality associated with hyperkalemia is not negligible, it may be important to educate patients about their susceptibility to hyperkalemia in winter.

The prevalence of hyperkalemia reported in the ED varies among studies from 0.3% to 13.3%, depending on the definition of hyperkalemia [1,2,3,4,5]. In our study, the prevalence of hyperkalemia was 6.6%, which is similar to previous studies. There are a variety of known risk factors for hyperkalemia [32,33,34]. Patients with reduced renal function are more likely to develop hyperkalemia [34]. In our study, the prevalence of hyperkalemia was significantly higher in patients with reduced renal function than in patients with preserved renal function. Also, it has been reported that the prevalence of hyperkalemia is higher in older age groups [32]. In this study, the prevalence of hyperkalemia was significantly higher in the elderly than in the young. The median age of the patients in our study was 73 years (56–81 years), which is relatively old, which may have led to the relatively high prevalence of hyperkalemia in our study.

This study has limitations. First, due to the single-center nature of the study, there is a possibility of selection bias in the patients enrolled. Second, several potential confounders were not collected, such as reason for visit, dietary noncompliance, medications that may cause hyperkalemia, and past medical history.

## 5. Conclusions

We found that the prevalence of hyperkalemia was significantly higher in winter for both patients with preserved renal function and those with reduced renal function.

## Figures and Tables

**Table 1:** Patient Characteristics.

	All	Preserved Renal Function	Reduced Renal Function
*n*	24,085	12,979	11,106
Male (%)	12,708 (53%)	6410 (49%)	6298 (57%)
Age (years)	73 (56–81)	65 (42–77)	78 (70–84)
Serum creatinine (mg/dL)	0.8 (0.6–1.2)	0.7 (0.6–0.8)	1.2 (1.0–2.1)
eGFR (mL/min/1.73 m^2^)	63 (42–83)	81 (70–98)	40 (22–51)
Prevalence of hyperkalemia (%)	6.6	2.1	11.9

Continuous variable is expressed as median (interquartile range).

**Table 2:** Seasonal Prevalence of Hyperkalemia.

	Spring	Summer	Autumn	Winter	*p*
Preserved renal function	2.3%	1.1%	2.0%	2.9% *	<0.001
Reduced renal function	12.5%	9.7%	10.8%	14.4% **	<0.001

* *p* < 0.001 vs. summer; ** *p* < 0.001 vs. summer and autumn.

## Data Availability

Upon reasonable request, the dataset used in this study is available from the authors.
